# Risk Factors and Prevalence of *Salmonella* spp. in Poultry Carcasses in Slaughterhouses Under Official Veterinary Inspection Service in Brazil

**DOI:** 10.3390/ani15162377

**Published:** 2025-08-13

**Authors:** Anna Carolina Massara Brasileiro, Cláudia Valéria Gonçalves Cordeiro de Sá, Carla Susana Rodrigues, Adriana Oliveira, Rafael Nicolino, João Paulo Amaral Haddad

**Affiliations:** 1 Department of Preventive Veterinary Medicine and Epidemiology, Veterinary College, Federal University of Minas Gerais, Belo Horizonte 31270-901, Brazil; 2 CIVG—Vasco da Gama Research Center/EUVG—Vasco da Gama University School, 3020-210 Coimbra, Portugal; 3 Department of Support and Standards, Ministry of Agriculture, Livestock and Food Supply, Secretariat of Animal and Plant Health and Inspection, Brasília 70043-900, Brazil

**Keywords:** *Salmonella*, chicken, risk characterization, epidemiology, Brazil

## Abstract

Because of the relevance of *Salmonella* spp. in poultry, in 2016, the Brazilian Ministry of Agriculture, Livestock, and Supply updated its regulation for the control and monitoring of *Salmonella* in the poultry meat production chain. We herein present the results of the official monitoring of sampling cycles of carcasses performed by the Brazilian official veterinary inspection service (SIF) and the Center for Epidemiology, Statistics, and Public Health of the Veterinary School of the Federal University of Minas Gerais. Samples were collected in March–September 2017, during which 140 slaughterhouses slaughtered over three billion chickens. The establishments were classified according to the number of animals slaughtered per day, and cycles varied according to the size of the establishment. Sample weights were defined to increase the external validity of the data. Chicken carcasses were randomly sampled after chilling and dripping. A total of 1434 samples were analyzed in official laboratories using Vidas SLM and SPT systems, for positive samples were used the ISO 6579:2014. The estimated prevalence of *Salmonella* spp. in chicken carcasses was 17.88% (95% CI 14.34–22.05). Considering the representativeness of Brazilian chicken meat in the global market, constant evaluation and review of the program is necessary to reduce the prevalence of *Salmonella*.

## Simple Summary

*Salmonella* is a type of bacteria that can cause serious foodborne illness in humans, and poultry meat is one of the main sources of this infection. To better protect consumers, the Brazilian government updated its rules in 2016 to improve how *Salmonella* is monitored and controlled in chicken production. This study presents the results of an official nationwide survey carried out in 2017. During this period, 140 slaughterhouses across Brazil were studied, and over 1400 chicken carcass samples were collected and analyzed in a presumptive detection method after processing. These samples were taken in a way that ensured they fairly represented the national production. The results showed that nearly 18% of the chicken carcasses were contaminated with *Salmonella*. This finding highlights the importance of maintaining strict control programs and regularly evaluating them to reduce contamination risks. Since Brazil is one of the world’s largest exporters of chicken, improving food safety helps protect not only national consumers but also millions of people around the world. This research supports the need for continued efforts in monitoring and prevention to ensure safer poultry products for everyone.

## 1. Introduction

Public health is globally affected by *Salmonella* spp. infections. This bacterial species uses a wide variety of animals as reservoirs and easily survives in various environments, hampering its control [1,2,3,4,5,6,7]. Broilers are considered one of the main reservoirs of *Salmonella*, with these animals and their derived products being important carriers of the pathogen [8,9,10,11]. Over 2600 *Salmonella* serovars are currently recognized, which mostly affect poultry. These serovars can basically cause two diseases: typhoid *Salmonella* (caused by *S*. *pullorum* and *S*. *gallinarum*) and nontyphoid *Salmonella* (several other serovars). These diseases may or may not be species-specific, as is the case with nontyphoid *Salmonella* that affects humans [8,12,13,14,15,16,17,18,19,20,21]. Expenses related to human infections by nontyphoid *Salmonella* have reached USD 3.65 billion annually in the European Union (EU) [22,23] and USD 3.7 billion in the United States of America (USA) [13]. A study on the costs for the Brazilian Unified Health System (SUS) of *Salmonella* spp. outbreaks linked to the consumption of animal products revealed an expense of USD 1.1 million [24].

The most commonly identified serovars in the EU and USA linked to human foodborne outbreaks are *S.* Enteritidis and *S.* Typhimurium. *Salmonella* Enteritidis is associated with the consumption of poultry meat and eggs, while *S*. Typhimurium is linked to a larger variety of foods, including pork, chicken, beef, and sheep [25,26,27,28,29,30,31]. In a study conducted in the USA in 2007–2011 [32], *S*. Enteritidis and *S*. Typhimurium serovars were correlated with several species of animals in the field, with clinical and non-clinical manifestations. Chickens also had the highest frequency (29.23%) of the *S*. Typhimurium serovar, but without clinical manifestation. Thus, integrating measures related to animal health in the field and processing industry are clearly necessary, as problems are identified in the phases of food processing, handling, transport, and storage, including in restaurants, supermarkets, and households. The risk inherent to food handling—the biggest challenge to mitigate the risks of *Salmonella* spp. infection [33,34,35,36,37,38]—is evident, as an erroneous conduct of the handlers leads to food contamination. In Brazil, most infections (when the place of contamination could be identified) occurred in residences (34.0%), restaurants, and bakeries (14.6%) [39]. Recently, China also identified differences between rural and urban cases of human infections [40].

In 2003, the Brazilian Ministry of Agriculture, Livestock and Supply (MAPA) established the Pathogen Reduction Program as a mitigation measure to monitor *Salmonella* spp. in chicken and turkey carcasses, providing a basis for more efficient control measures in the production process [41]. Based on exploratory studies, this program was revised and updated in 2016. The current standard was elaborated based on American [42] and European [43] regulations, in order to meet the requirements of both markets. In the current standard, animal health defense actions were integrated, covering the entire production chain (from farm to slaughterhouse). Chickens destined for slaughter must be accompanied by a laboratory report of the *Salmonella* spp. test; moreover, a positive result must be species-specified—i.e., for *S*. Enteritidis, *S*. Typhimurium, *S*. Gallinarium, *S*. Pullorum, and/or monophasic salmonela (1,4[5],12:-:1,2/1,4[5],12:i:-), the monophasic genomic variant of *S*. Typhimurium. Batches positive for *Salmonella* spp., *S*. Gallinarium, and *S.* Pullorum must be separately sacrificed, and the entire line immediately hygienized. Batches positive for *S*. Enteritidis, *S*. Typhimurium, and monophasic *Salmonella* must be separately slaughtered, equipment must be immediately sanitized, and the carcasses can be destined for mechanically separated meat and cooked products [44].

These preventive and control measures, in addition to verifying *Salmonella* spp. contaminations in poultry, are also applied to industrial self-control programs. These mitigation measures are also integrated with the production chain, making it more effective and promoting food safety for the consumer.

The objective of the present study was to determine the prevalence of *Salmonella* spp. in chicken carcasses, as well as to characterize the risk of contamination according to the size and commercial qualification of Brazilian slaughter establishments under the Brazilian veterinary inspection service in 2017. The findings from this investigation will serve as technical guidance for the official sanitary inspection service, aiming to enhance preventive control measures for improved public health protection.

## 2. Materials and Methods

A cross-sectional, nationwide prevalence study of *Salmonella* spp. was conducted on chicken carcasses slaughtered in slaughterhouses under the Brazilian official inspection service from March to September 2017. During the study period, 140 establishments slaughtered more than 3 billion chickens in the country. A total of 1434 samples from 115 slaughter establishments were analyzed in the Brazilian official Federal Laboratories for Agricultural Defense (LFDAs).

### 2.1. Sampling

Sampling methods were defined after the publication of the regulation, contemplating field mitigation measures [44], and were based on previous monitoring studies implemented by the Department of Inspection of Products of Animal Origin (DIPOA), with the support of members of the Scientific Advisory Committee on Microbiology of Products of Animal Origin [45]. Two-level sampling considering establishments and carcasses was used, and their respective sample weights were implemented to increase the external validity of the data [46,47]. To define the sampling plan, slaughterhouses under the Brazilian official veterinary inspection service were categorized into Small, Medium, Large, and Very Large, according to the number of chickens slaughtered per day (Table 1). 

### 2.2. Sample Collection

The data herein analyzed were obtained from the official collections of the veterinary service, and all days of the week and slaughter shifts were considered to have the same chance of being sampled. Samples (carcasses) were randomly collected immediately after the drip stage and before packing [44], and then sent refrigerated or exceptionally frozen to the official laboratories (LFDA) for *Salmonella* spp. testing. The criteria for defining the cycles and sampling were proportional to the sizes of the slaughter establishments (Table 2) [44,48]. Expected prevalence and probability were estimated based on previous studies.

A total of 1434 samples were collected from 115 establishments, 95 of them qualified for the foreign market (EM) and 20 qualified exclusively for the domestic market (IM) [49] (Table 3).

### 2.3. Microbiological Analysis

In the official laboratories, 25 g of skin and muscle from the pericloacal, wing, and neck regions of each carcass was collected. The presumptive detection method by enzyme immunoassay reaction (VIDAS^®^ Easy *Salmonella* Method (SLM), bioMérieux, Lyon, France, Validation: AFNOR BIO 16/12-09/05 or VIDAS^®^ UP *Salmonella* (SPT), Validation: AFNOR BIO 12/32-10/11) was used. Samples with positive results for *Salmonella* spp. were subjected to confirmatory analysis using the ISO 6579:2014 methodology, using specific O and H antisera according to the White–Kauffmann–Le Minor scheme for serological confirmation [50].

This method ensures standardized detection of *Salmonella* across various matrices, with validated performance characteristics, including sensitivity and specificity, as discussed by Mooijman [51]. All official laboratories involved in the analyses have the procedures controlled and accredited by ISO/IEC 17025 and ISO 6579:2014 [50,52].

### 2.4. Statistical Analysis

Sample collection and laboratory results data were stored in electronic spreadsheets. After verification and adjustments, the status of samples and establishments was characterized. Statistical analysis was performed based on the principle of probability, applicable in the definition of a sampling plan as required by the *Codex Alimentarius* [53]. Sample weights of the establishments were calculated based on production capacity and sampling unit, as follows:

$$\mathrm{Sample}\;\mathrm{weight}:\;\frac{total\;number\;of\;slaughterhouses}{number\;of\;slaughterhouses\;sampled}\times \frac{total\;production\;of\;the\;slaughterhouse\;sampled}{number\;of\;samples\;of\;the\;slaughterhouse\;sampled}$$

In addition to descriptive statistics, logistic regression models were used, considering the sample weights of establishments and samples. The dependent variable was *Salmonella* spp. results in slaughter establishments, and the independent variables were their respective sizes, qualifications, and the day of the week of collection. The weighting of the data for each production volume provides a scientific basis for the assessment of the exposure of products to the pathogen and their use in microbial risk evaluations. Model goodness-of-fit used was the Hosmer–Lemeshow test for logistic regression; the results include standard errors and 95% confidence. Sample collection data and laboratory results were analyzed using the Stata 15 statistical software (Stata Statistical Software: Release 15. College Station, TX: StataCorp LP, Texas, USA).

### 2.5. Geoprocessing

To perform the geoprocessing of slaughterhouses, we listed all active chicken slaughterhouses in 2017, consulting their respective addresses. All information was extracted from SIGSIF and MAPA’s website [49]. The Google Maps platform was then used to search for geographic coordinates, which were entered into the ArcGIS Pro Version 3.5 [54] for geoprocessing.

**Figure 1:**
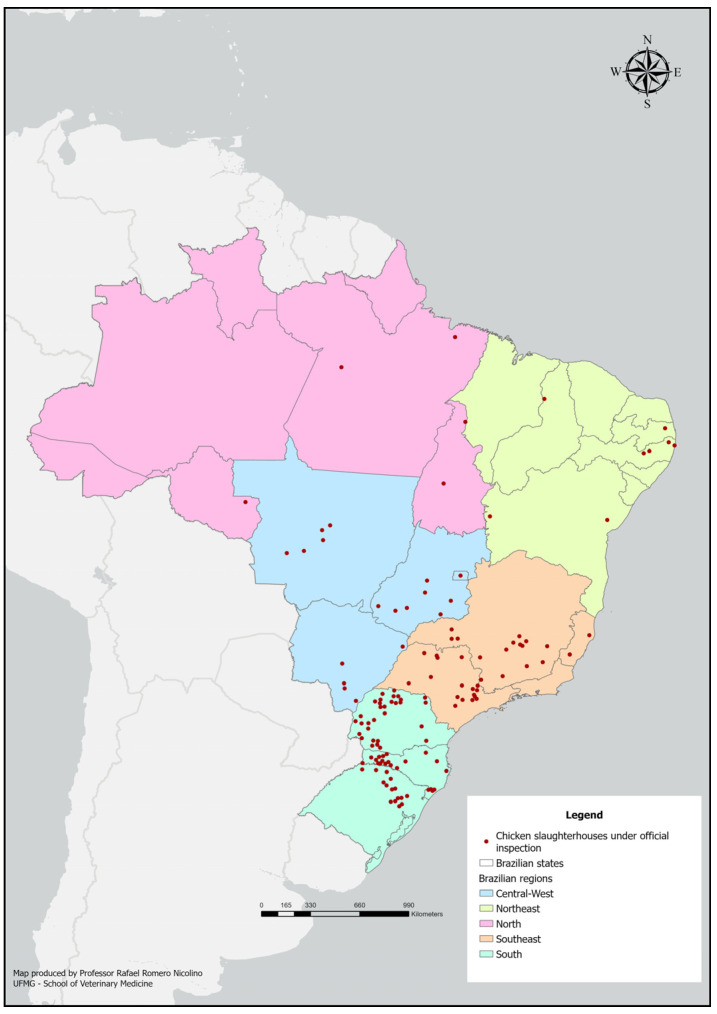
Spatial distribution of poultry slaughter establishments with samples collected under federal inspection service in Brazil, in 2017.

The distribution of Brazilian chicken slaughter establishments under the federal inspection service is shown in Figure 1.

## 3. Results

The prevalence of *Salmonella* spp. in broiler carcasses in Brazil was 17.88% (95% CI 14.34–22.05) of 20,484,000 carcasses. The prevalence in small slaughterhouses was 25.18% (95% CI 14.41–40.20), which was not significantly different from that of the other slaughterhouse sizes (Table 4).

Considering the day of collection, the highest prevalence was on Monday (21.74%), and Thursday presented a significant protection factor (0.29; 95% CI 0.14–0.58; *p* = 0.001) compared to the other days of the week. However, since the self-control programmes are standardized and, in the present study, there is no information about working shifts and workers’ presence, factors that could influence these results, further investigation is necessary to check the possible associated reasons.

Most slaughter establishments were concentrated in the South (52.14%) of Brazil, followed by the Southeast (25.71%), Center-West (13.57%), Northeast (5.71%), and North (2.86%). The regional characterization of *Salmonella* prevalence in slaughter establishments was similar among these regions; the highest prevalence was in the South, followed by the Midwest, Southeast, and North/Northeast regions of Brazil (Table 5).

**Table 5:** Regional characterization of *Salmonella* spp. prevalence in chicken carcasses in Brazilian slaughterhouses under official veterinary inspection service in 2017.

Region	No. of Slaughter Establishments Sampled	% *Salmonella*	CI (95%)
South	63	18.42	1.38–24.14
Central-west	11	17.48	10.35–27.98
Southeast	33	16.58	10.5–25.19
North/Northeast	8	16.05	0.51–40.31

*Salmonella* spp. prevalences in slaughterhouses with different marketing qualifications, for which no statistically significant difference was found, are shown in Table 6.

**Table 6:** Prevalence of *Salmonella* spp. in relation to the commercial scope.

Commercial Qualification	% *Salmonella*	CI (95%)
IM	19.59	8.5–39.9
EM (European Union)	18.82	12.7–26.9
EM (Other)	16.98	13.1–21.8

IM, Domestic Market; EM, External Market.

The five main serovars identified in the positive samples are shown in Table 7.

**Figure 2:**
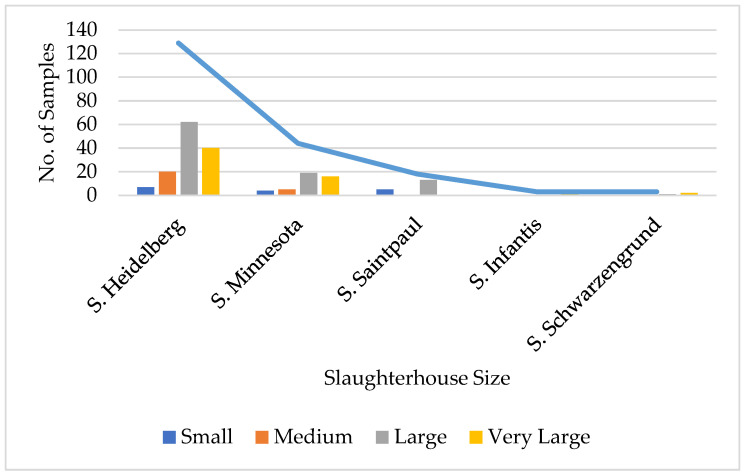
*Salmonella* serovars according to slaughterhouse size.

The distribution of the five main *Salmonella* serovars according to slaughterhouse size is shown in Figure 2.

## 4. Discussion

### 4.1. Brazilian Prevalence Comparison with Other Countries

The prevalence of *Salmonella* spp. in chicken carcasses in Brazil in the study period was 17.88% (95% CI 14.34–22.05), which is lower than the value found in a study conducted in the USA in 2012. However, the latter study evaluated parts of carcasses and viscera in which the prevalence was 24.02% (95% CI 19.24–28.79). Prevalence was compared between skinned and skinless samples, but no significant difference was detected [42]. The carcass cutting process exposes muscle surfaces that are particularly vulnerable to bacterial contamination, especially in the presence of pre-existing surface contamination [55,56]. A baseline study conducted in the EU in 2008 obtained results similar to those from Brazil, with a prevalence of 15.6% (95% CI 13.6–17.9) in neck and breast skin samples. Variation among member state countries (MS) was detected, with Hungary (85.6%), Bulgaria (26.6%), Poland (25.4%), and Slovakia (22.8%) standing out. The other countries did not show such disparity in prevalence, and Denmark, Estonia, Finland, Luxembourg (MS), and Norway (non-MS) showed negative results for *Salmonella* in chicken carcasses [57].

The microbiological acceptance standards of the cycles were based on studies developed in previous years and were established according to each reality, as recommended in the *Codex Alimentarius* [53] and international microbiological standards [48]; this allowed the Brazilian *Salmonella* control program to be standardized with those of countries that have more stringent sanitary rules. Cycle variation is proportional to the size of the slaughterhouse and was calculated by estimating a prevalence of 20% [44]. The expected prevalence in the EU and in the USA is approximately 11.3% (*n* = 50; c = 7) [43] and 7.5% (*n* = 51; c = 5) [58], respectively.

The creation of the National Pathogen Control Program (PNCP) in 2013 allowed the evaluation of the frequency of *Salmonella* spp. in chicken carcasses. In the 2013–2014 cycle, 856 samples were collected from 89 establishments, obtaining a prevalence of 17.52%. In 2015–2016, 1922 samples from 143 establishments were analyzed; 330 samples had positive results, resulting in a frequency of 17.17% [59]. A progressive fluctuation in the occurrence of *Salmonella* spp. in poultry carcasses was observed between 2017 and 2022, with an initial decline followed by subsequent increases. Between 2018 and 2022, the occurrence of *Salmonella* spp. in poultry carcasses exhibited moderate fluctuations, with rates ranging from 12.61% to 14.86%, indicating slight variations over time but no consistent downward trend [59]. However, it can be observed that in these most recent results, in Brazil, *Salmonella* occurrence is lower than that presented in the current study, indicating the effectiveness of the measures adopted to ensure public health protection.

### 4.2. Slaughterhouse Size

No statistically significant difference in prevalence was found among slaughter establishments of different sizes; however, constant monitoring should be maintained, as small-sized slaughter establishments tend to have a higher prevalence than other establishment sizes. A prevalence study conducted in France in 2008 on the risk factors influencing the presence of *Salmonella* in carcasses found that establishments slaughtering 50,000–150,000 animals per day had a high percentage of positive carcasses. The specific risk factors were the number of operators present at evisceration (<2) (OR = 4.65) and the exclusivity of the establishment in slaughtering only *Gallus* (OR = 7.08) [60]. Algeria also reported a high *Salmonella* prevalence in small-scale establishments [61].

### 4.3. Regional Influence

No statistically significant differences in prevalence were found among the regions of Brazil. This may indicate that the quality standards of the regions are uniform in the field and industry, despite regional challenges such as the climate. Nevertheless, previous studies in different regions detected some differences. In Rio Grande do Sul, in 2006–2015, the average prevalence of *Salmonella* spp. in 77,165 analyzed carcasses was 4.04% [62]; and in the state of São Paulo in 2000–2010, 609 samples had a prevalence of 14.6% [63]. However, these studies used different methodologies. Analysis of USDA-FSIS demonstrated regional variation in serovar distribution, with *S.* Infantis and *S.* Typhimurium predominating in the Atlantic and *S.* Schwarzengrund in the Southeast. In Georgia, comparison between processing and breeder flock data indicated *S.* Kentucky as the dominant serovar in breeders (67.91%), though largely absent at processing, likely due to effective antimicrobial interventions [64]. These findings highlight the importance of implementing region-specific serovar surveillance in Brazil to better inform control strategies and improve public health outcomes.

### 4.4. Operational Influences

Considering the day of collection, the highest prevalence was on Monday (21.74%), and Thursday presented a significant protection factor (0.29; 95% CI 0.14–0.58; *p* = 0.001) compared to the other days of the week. However, as the self-control programs are standardized, and the present study lacks information regarding operational details, factors that could potentially influence the results [55,65,66,67]. Further investigation is required to identify the underlying causes associated with the observed outcomes.

Ensuring product safety requires not only standardized processing but also strict biosecurity measures at the farm level. Key practices include sourcing *Salmonella*-free chicks, using pelleted feed with organic acids, maintaining water quality, effective pest control, and regulated access to facilities [68,69,70,71,72]. Although poultry slaughter is automated, pathogen control depends heavily on well-implemented self-control programs focused on sanitation and hygiene [73,74,75,76]. Critical control points—starting with animal welfare and continuing through pre-slaughter handling, scalding, evisceration, and chilling—must be carefully managed to reduce stress and microbial load [73,77,78,79,80,81,82,83,84]. Notably, *Salmonella* has been detected even after cage sanitization, highlighting the importance of rigorous cleaning protocols to prevent farm-to-plant pathogen transmission [73,74,77,78,79,80,81,82,83,84].

### 4.5. Prevalence and Serotyping According to Establishments Qualification

In 2017, 66.9% of production was destined for domestic consumption and 33.1% for the foreign market [85]. Regarding the minimal and non-significant difference in prevalence among establishments with different commercial qualifications, it is important to highlight that the very large and large establishments are export-oriented establishments. They are officially certified for international trade and also operate in the domestic market. These establishments are responsible for a substantial portion of the domestic supply due to their high production volume. The main destinations of national poultry meat are countries in the Middle East, Asia, and Africa [86].

The five main serovars found in this study—*S*. Heidelberg (9.0%), *S*. Minessota (3.07%), *S.* Saintpaul (1.2%), *S.* Infantis (0.21%), and *S*. Schwarzengrund (0.21%)—were also found in previous studies conducted in Brazil. Enteritidis and Typhimurium serovars were identified in 0.25% and 0.51%, respectively, in 2017 b, demonstrating the effectiveness of the official national vaccination program instituted in the field since 2003 [41]. In a field prevalence study conducted in 2009–2010 in the states of Paraná, Santa Catarina, and Mato Grosso do Sul to identify the main serovars circulating in broiler production, the most frequent serovars identified from 1543 samples were Minnesota (40.24%), Infantis (14.63%), Heidelberg (7.31%), Senftenberg (6.09%), and Mbandaka (6.09%). Between 2007 and 2011, 12,582 strains of *Salmonella* from chicken carcasses and products from all regions of the country were analyzed by the Oswaldo Cruz Foundation. Sixty-one different serovars were identified, among which the main ones (53%) were Enteritidis, Minnesota, Typhimurium, Schwarzengrund, and Mbandaka. The Heidelberg serotype was among the 20 most frequent in the study and had not yet been isolated in the southern region [87]. We herein show that it is currently notably present in the southern region.

### 4.6. Public Health Impact

According to the Brazilian government [88], in 2017, 10 outbreaks caused by *Salmonella* spp. were reported, resulting in 378 cases, along with one outbreak attributed to *Salmonella* Typhimurium, which resulted in 15 cases. None of these outbreaks were linked to the consumption of chicken meat or derived products. The serovars *S*. Heidelberg, *S*. Senftenberg, and *S*. Mbandaka have been identified in broilers for years, but there are no studies establishing their relationship with foodborne outbreaks [8]. More recently, in Brazil, among the *Salmonella* spp. isolates subjected to serotyping, *S.* Minnesota and *S.* Heidelberg were the most frequently identified serotypes, accounting for 60% and 21.7% of samples, respectively. Serotypes of greater public health concern, such as *S.* Typhimurium and the monophasic variant *S.* 1,4,[5],12:i:-, both of which are classified within the same serovar, Typhimurium, were detected at low frequency (0.48%), while no isolates of *S.* Enteritidis were identified [59]. Although the health status of live animals was not assessed in this study, it is important to emphasize that all animals testing positive for *Salmonella enterica* serovars Typhimurium or Enteritidis are systematically slaughtered separately from other flocks, at the end of the processing shift. Their carcasses are subsequently subjected to heat treatment to ensure effective elimination of the pathogen.

In addition to the challenge of mitigating *Salmonella*, there remains the significant concern of antimicrobial resistance (AMR) and its public health implications. In Brazil, the National Action Plan for the Prevention and Control of Antimicrobial Resistance in Animal Production was established in 2018. The plan aims to promote awareness, monitoring, and control of antimicrobial use and resistance across multiple areas of intervention [89]. As part of these efforts, the MAPA conducted an exploratory study on antimicrobial resistance in *Salmonella enterica* from poultry meat, involving the analysis of 146 isolates from 2014 and 163 from 2017. *Salmonella* ser. Heidelberg and *Salmonella* ser. Minnesota were the main serotypes associated with resistance. Multidrug resistance was detected in 50.7% (74/146) of isolates in 2014 and increased significantly to 77.3% (126/163) in 2017 (*p* < 0.05). The most frequent resistance profiles were observed against nalidixic acid (57.5% and 86.5%), ampicillin (56.2% and 76.7%), cefotaxime (52.1% and 76.1%), ceftazidime (50.0% and 76.1%), ciprofloxacin (56.9% and 89.0%), and tetracycline (60.3% and 82.8%) for 2014 and 2017, respectively [90]. In a recent study conducted in the UK, *Salmonella* strains originating from Brazilian poultry products are unlikely to cause disease in UK consumers. No evidence of *Salmonella Heidelberg* or *Salmonella Minnesota*—serovars detected in Brazilian poultry—was found in UK domestic chicken populations. These results indicate that the introduction of the *Salmonella Enteritidis* vaccine in Brazil, along with increased antimicrobial use, may have led to the replacement of more virulent serovars with those exhibiting higher antimicrobial resistance but lower pathogenicity in humans within the UK context [91].

A study conducted in the USA on the incidence rates of *Salmonella* serovars associated the Saintpaul (2%), Heidelberg (2%), and Infantis (1.9%) serovars with most foodborne infections from 2010 to 2015. Enteritidis (18%), Newport (10.7%), and Typhimurium monophasic serovars (9.8%) are the most representative [25]. It should be noted that the previous study does not detail the types of products linked to the outbreaks. In 2012, the most prevalent serovars in chicken carcasses and cuts were Kentucky (30.0%), Enteritidis (24.7%), Typhimurium monophasic (20.55%), Heidelberg (7.6%), and Thompson (4.3%) [42]. In another study conducted in the USA in 2013, the Heidelberg serotype was associated with a foodborne outbreak due to the consumption of contaminated chicken cutlets, affecting 634 people [10]. In 1970–2009, the Heidelberg serotype was among the most common in chickens and humans [92].

In the EU, 77,486 cases of human salmonellosis have been confirmed in all member states. The serovars most commonly identified and associated with cases were *S*. Enteritidis (70.8%), *S*. Typhimurium (8.9%), monophasic *S*. Typhimurium 1,4,[5],12:i:- (5.1%), *S*. Infantis (2.0%), and *S*. Coeln (0.77%). *Salmonella* Enteritidis was associated with hen eggs and broiler meat, while *Salmonella* Typhimurium was associated with pork consumption, and *S*. Infantis was strongly associated with chicken consumption [93].

The Minnesota serovar is rarely associated with foodborne outbreaks worldwide [8,92,94].

### 4.7. Efforts Improvements on Surveillance

The continuous evaluation and refinement of pre-harvest and post-harvest interventions are essential for adapting to emerging challenges, such as evolving pathogen profiles and changing production practices. Studies focusing on innovative interventions and technologies intended to complement the established approaches will be crucial in mitigating microbial evolution [68,95,96].

Recently, China applied whole-genome sequencing and machine learning to characterize *Salmonella* from different retail chicken supply modes, revealing distinct genetic profiles, serotypes, and resistance patterns. Advanced genomic analyses identified clonal persistence and critical resistance genes, particularly in frozen products [97]. These findings underscore the value of integrating emerging technologies—such as machine learning and microbiome analysis—into food safety monitoring systems to enhance pathogen surveillance and strengthen multi-hurdle control strategies against *Salmonella* in poultry production.

## 5. Conclusions

The size of the slaughterhouses, their respective markets, and location did not affect *Salmonella* prevalence. However, we could identify the serovars present in Brazilian slaughterhouses and their association with possible foodborne outbreaks. Additionally, attention is given to the identification of *Salmonella* serovars Heidelberg and Infantis in field-derived reports, with measures such as product segregation and restriction of their use exclusively to cooked products, due to their potential to cause human salmonellosis. Our data prove the need for constant monitoring of the surveillance program. Improvements and more detailed studies can be planned considering a greater number of variables, for example, further investigation into the observed trend of higher contamination rates on Mondays, and the use of technologies, aiming at food safety and risk mitigation by the Brazilian official veterinary service.

## Figures and Tables

**Table 1:** Size of Brazilian chicken slaughterhouses under official veterinary inspection service according to slaughter volume.

Port	No. of Poultry Slaughtered/Day	No. of Slaughterhouses Analyzed
Small	<50,000	16
Medium	50,001–100,000	30
Large	100,001–200,000	44
Very Large	>200,001	25

Source: Normative Instruction No. 20 [44].

**Table 2:** Distribution of the official sampling plan of chicken carcasses according to the size of the slaughterhouses. Expected prevalence 20%, probability 80%.

Slaughterhouse	*n*	c	No. of Cycles/Year	Collection Frequency
Small	8	2	2	1 sample/3 weeks
Medium	8	2	2	1 sample/3 weeks
Large	8	2	2	1 sample/2 weeks
Very Large	8	2	2	1 sample/2 weeks

*n*: number of samples taken, c: maximum number of acceptable positive samples, No.: number. Source: Normative Instruction No. 20 [44].

**Table 3:** Samples of chicken carcasses collected according to the scope of commercialization, Brazil, 2017.

Marketplace	No. of Slaughtering Establishments	No. of Slaughter Establishments Sampled	No. of Samples Analyzed
External (EM)	116	95	(1267)
Internal (IM)	24	20	(167)
Total	140	115	(1434)

**Table 4:** Prevalence of *Salmonella* spp. considering the size of Brazilian chicken slaughterhouses under official veterinary inspection service in 2017.

Slaughterhouse Size	% *Salmonella*	Number of Positive Samples	CI (95%)
Small	25.18	25 (124)	14.41–40.20
Medium	17.15	42 (256)	9.8–28.35
Large	18.7	114 (633)	13.75–24.97
Very Large	17.13	68 (421)	11.93–23.97

CI = confidence interval.

**Table 7:** Five main *Salmonella* serovars identified in chicken carcasses from slaughterhouses under Brazilian official veterinary inspection service in 2017.

*Salmonella Serovars*	Number of Isolates	%
*Salmonella* Heidelberg	129	9.0
*Salmonella* Minnesota	44	3.07
*Salmonella* Saintpaul	18	1.26
*Salmonella* Infantis	3	0.21
*Salmonella* Schwarzengrund	3	0.21

## Data Availability

The data presented in this study are available on request from the corresponding author due to official confidentiality restrictions. These data were provided by the Brazilian Ministry of Agriculture, Livestock and Food Supply (MAPA) and are subject to prior authorization for use, as detailed in the attached official approval document (Process No. 21000.048282/2020-58).

## Abbreviations

The following abbreviations are used in this manuscript:

CI	Confidence Interval
DIPOA	Department of Inspection of Animal Products
EM	External Market
EU	European Union
IM	Internal Market
ISO	International Organization for Standardization
LFDAs	Federal Laboratories of Agriculture Defense
MAPA	Ministry of Agriculture, Livestock and Food Supply
MS	Member State
NM	National Market
No.	Number
SDA	Secretariat of Animal Defense
SUS	Unified Health System
UFMG	Federal University of Minas Gerais
USA	United States of America
